# Breath Prints for Diagnosing Asthma in Children

**DOI:** 10.3390/jcm12082831

**Published:** 2023-04-12

**Authors:** Valentina Sas, Paraschiva Cherecheș-Panța, Diana Borcau, Cristina-Nicoleta Schnell, Edita-Gabriela Ichim, Daniela Iacob, Alina-Petronela Coblișan, Tudor Drugan, Sorin-Claudiu Man

**Affiliations:** 1 Department of Pediatrics, “Iuliu Hațieganu” University of Medicine and Pharmacy Cluj-Napoca, 400124 Cluj-Napoca, Romania; tarausasvalentina@gmail.com (V.S.);; 2 Clinical Hospital for Pediatric Emergencies, 400124 Cluj-Napoca, Romania; 3 Department of Nursing, “Iuliu Hațieganu” University of Medicine and Pharmacy Cluj-Napoca, 400124 Cluj-Napoca, Romania; 4 Department of Medical Informatics and Biostatistics, “Iuliu Hațieganu” University of Medicine and Pharmacy Cluj-Napoca, 400124 Cluj-Napoca, Romania

**Keywords:** electronic nose, asthma, breath analysis, volatile organic compounds

## Abstract

Electronic nose (e-nose) is a new technology applied for the identification of volatile organic compounds (VOC) in breath air. Measuring VOC in exhaled breath can adequately identify airway inflammation, especially in asthma. Its noninvasive character makes e-nose an attractive technology applicable in pediatrics. We hypothesized that an electronic nose could discriminate the breath prints of patients with asthma from controls. A cross-sectional study was conducted and included 35 pediatric patients. Eleven cases and seven controls formed the two training models (models A and B). Another nine cases and eight controls formed the external validation group. Exhaled breath samples were analyzed using Cyranose 320, Smith Detections, Pasadena, CA, USA. The discriminative ability of breath prints was investigated by principal component analysis (PCA) and canonical discriminative analysis (CDA). Cross-validation accuracy (CVA) was calculated. For the external validation step, accuracy, sensitivity and specificity were calculated. Duplicate sampling of exhaled breath was obtained for ten patients. E-nose was able to discriminate between the controls and asthmatic patient group with a CVA of 63.63% and an M-distance of 3.13 for model A and a CVA of 90% and an M-distance of 5.55 for model B in the internal validation step. In the second step of external validation, accuracy, sensitivity and specificity were 64%, 77% and 50%, respectively, for model A, and 58%, 66% and 50%, respectively, for model B. Between paired breath sample fingerprints, there were no significant differences. An electronic nose can discriminate pediatric patients with asthma from controls, but the accuracy obtained in the external validation was lower than the CVA obtained in the internal validation step.

## 1. Introduction

Asthma continues to be one of the main concerns of pediatricians. It is one of the most frequent chronic diseases in childhood with an increasing prevalence in developed countries, and its management incurs a high cost. According to the GINA consensus, the clinical aspects, pulmonary function test and response to bronchodilator treatment can indicate a diagnosis of asthma. Symptoms are variable and pulmonary function tests may be difficult to perform in small children [1,2,3,4]. The standardized diagnostic methods for asthma assess the reversible obstruction in the airway rather than the inflammation, which is part of the pathological mechanism of asthma [5]. The research in this field has helped develop new diagnostic tools to distinguish between different inflammatory phenotypes, such as the identification of eosinophils and neutrophils in induced sputum and the measurement of nitric oxide (FeNO) in the exhaled breath. Induced sputum and provocation tests are difficult to obtain in children and require time. Only the measurement of FeNO from the exhaled breath has proven to be useful and easy to perform [6]. 

Exhaled breath contains volatile organic compounds as a result of metabolism; the composition of VOC is modified by respiratory and non-respiratory diseases. The analysis of VOC’s composition can identify specific profiles for each disease, making the diagnosis more accurate. The gold standard for detecting VOC’s composition is mass spectrometry or chromatography, but these methods are expensive and are not readily accessible [7,8]. 

The use of an e-nose for analyzing the exhaled breath composition is an innovative technique in the field of medicine. It can be the key to a quick diagnostic method for many acute or chronic diseases. Studies in this field have shown promising results regarding the ability of e-nose to recognize diseases; however, it is necessary to perform more clinical trials so that the technique can be validated and used in clinical practice [9,10,11,12,13,14,15,16]. 

In medicine, e-nose has been studied so far, especially in adults, for diseases, such as asthma [9,17], lung cancer [18,19,20], tuberculosis [21,22,23], chronic obstructive pulmonary disease (COPD) [24] and interstitial lung disease [25]. In pediatrics, the method has been applied for the diagnosis of asthma, cystic fibrosis, primary ciliary dyskinesia [10,11,13,14,15,26] and obstructive sleep apnea [27]. So far, e-nose has proved to be useful in recognizing the exhaled breath pattern of patients with asthma, but it is less accurate in identifying the severity. The VOC’s composition in the exhaled breath sample does not change in asthma exacerbations and is not related to the airway caliber [17,28]. Brinkman et al. demonstrated that e-nose can identify asthma phenotypes that correlate with age and symptoms variability, suggesting that this aspect may be the key to individualized treatment [13]. A recent study published in 2020, performed by Tenero et al., showed that e-nose could correctly distinguish controls from asthmatic pediatric patients with different severity [14].

**Figure 1:**
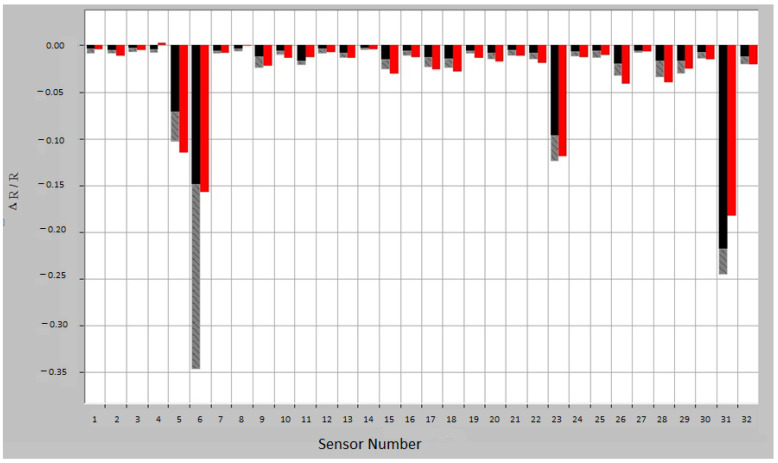
Breath sample fingerprint provided by e-nose for a patient with asthma. ΔR/R = (R_max_ − R_0_)/R_0_, where R_0_ is the resistance during a baseline gas flow and R_max_ is the maximum resistance during exposure to the sample. Red—sample response; Training response variation: black—minimum exposure and gray—maximum exposure.

E-nose consists of an array of nanosensors with different selectivity, a signal pre-processing unit and a pattern recognition unit. Compared with mass spectrometry or chromatography, e-nose does not identify specific chemical components. The VOC contained in the exhaled breath sample interacts with the array of nanosensors, modifying their electrical resistance; the sensor response is measured as (R_max_ − R_0_)/R_0_, where R_0_ is the resistance during a baseline gas flow and R_max_ is the maximum resistance during exposure to the sample. The raw sensor response, converted into a value, is then introduced into the data analysis system, creating a characteristic fingerprint, which can be analyzed by the recognition system (Figure 1).

Cyranose 320, a handheld and portable device, is the e-nose device provided by Sensigent. Currently, the Cyranose 320 is used in diverse industries, including petrochemical, chemical, food and beverage, packaging materials, plastics, pet food, pulp and paper, medical research and many more [29].

In pediatrics, it is very important to use non-invasive diagnostic tools to avoid discomfort to patients. E-nose is easy to perform in children and is an innovative, simple, quick and inexpensive technique; the device is portable, easy to use and well accepted by the patients. This study aimed to evaluate the diagnostic accuracy of e-nose for asthma in children. For this, we hypothesized that e-nose can differentiate between asthma and control patients. To test this hypothesis, our objectives were to evaluate the accuracy of two classification models (asthma vs. controls), to test the external validation of these models and to evaluate the reproducibility of the measurements.

## 2. Materials and Methods

Subjects. We included 35 patients, aged 5 to 18 years, who were evaluated in the Third Pediatric Clinic of the Clinical Hospital for Pediatric Emergencies from Cluj-Napoca, Romania, between 1 February and 30 June 2020. The study design had two steps. First, there was the training stage and then the validation stage of the obtained models on a new set of patients. The study group included children diagnosed with asthma and the control group included children assessed in the hospital for non-respiratory chronic diseases. The training set included 18 patients, 11 cases and 7 controls, which formed the two training models A and B. The validation set included 9 cases and 8 controls.

Inclusion criteria. Asthma patients: aged between 5 to 18 years, diagnosis of asthma at least 6 months before and no past medical history of chronic disease or acute respiratory tract infection in the last 4 weeks. Controls: aged between 5 to 18 years, no respiratory disease, no past medical history or family history of atopy, recurrent wheezing or asthma and no acute respiratory tract infection in the last 4 weeks. We used the same inclusion criteria for patients and controls in the training models and in the validation set.

For the diagnosis of asthma, we considered GINA consensus criteria—a history of variable respiratory symptoms: recurrent episodes of wheezing, breathlessness, chest tightness and cough, especially at night or during the early morning; the presence of symptoms induced by physical activity, allergen exposure, cold air, laughing, or during viral infections; and also lung function test criteria [5]. Regarding the spirometry, we considered reversible airway obstruction when FEV1 was less than 80% of the predictive value, FEV1/FVC was decreased and FEV1 increased by more than 12% of the predictive value after 400 µg inhaled salbutamol. 

Patients and caregivers signed the informed consent, and the study was approved by the Ethics Committee of the “Iuliu Hațieganu” University of Medicine and Pharmacy from Cluj-Napoca, Romania, no. 359 of 28 September 2017.

Breath sample. In the absence of a standard protocol for the collection of exhaled breath samples, we used the protocol proposed by Dragonieri et al. [17]; the measurements were made in the same room and in the same ambient conditions (temperature and humidity). We used a silica filter to limit the influence of humidity on the sensor’s signal. The manufacturer recommends disabling sensor numbers 5, 6, 23 and 31, which are the most affected by humidity. In our case, disabling the sensors did not change the accuracy rates.

The test was performed for each patient; they were not allowed to eat and drink two hours before the test and it was contraindicated to take inhaled medication or oral antihistaminic drugs 12 h before the test. The patients were asked to breathe 5 times into a device with a VOC filter from ambient air. After a forced inspiration through this device, the patient exhaled a volume of air, equal to their vital capacity, into a 5 L Tedlar sampling bag. The sample from the Tedlar bag was then connected to the e-nose device (Cyranose 320). The changes in the resistance of the nanosensors were stored as primary data, which were then analyzed by the recognition system [17].

Data analysis. The study had a cross-sectional case-control design. The exhaled air was first analyzed online by the onboard learning software of the Cyranose 320 (PCNose). The raw sensor response was converted into a value, as described above, and then introduced into the data analysis system. Secondly, the results were confirmed offline by the CDAnalysis program, version 11.2, through the double cross-validation implementation of canonical discriminant analysis on the principal component reduction using Matlab software. The data were processed through Savitzky–Golay filtering and baseline correction, based on the recommendations of the manufacturer. Using PCA, the data from the 32 sensors were reduced to a set of principal components. PCA generated two- and three-dimensional graphs, visualizing the differences between groups. For the construction of the recognition algorithm, canonical discriminative analysis (CDA) was performed to maximize the distance between groups. The online software calculated a cross-validation value (CVV) while the offline software calculated cross-validation accuracy (CVA). M-distance quantifies the discrimination between groups. An M-distance of more than 3 was indicative of a high probability of discrimination. To compare the clinical characteristics, Fisher, chi-square, ANOVA and t-test were used. For the diagnostic performance of the training models in the external validation phase, we used the uncorrected chi-square Pearson test, Yates test, Mantel Haenszel test and Fisher test. Pearson’s correlation coefficient was calculated to evaluate the breath prints’ reproducibility.

## 3. Results

There were 18 patients in the training models divided into two smaller sets: model A with 11 subjects (6 with asthma and 5 controls); and model B with 12 patients (5 with asthma and 7 controls). Five of the seven controls in model B were the same as those in model A. For the external validation set, we had 17 subjects: 9 patients with asthma and 8 controls. Table 1 and Table 2 describe the characteristics of the patients. Patients with asthma were younger than controls in model A. In the study, there were 12 girls (8 in the training models and 4 in the external validation set) and 23 boys (10 in the training models and 13 in the external validation set).

Table 2 lists the clinical characteristics of asthmatic patients. More than half of the patients had atopy, high total IgE levels and positive specific IgE to airborne allergens. Asthma was partially controlled in most of them.

### 3.1. Internal Validation

**Figure 2:**
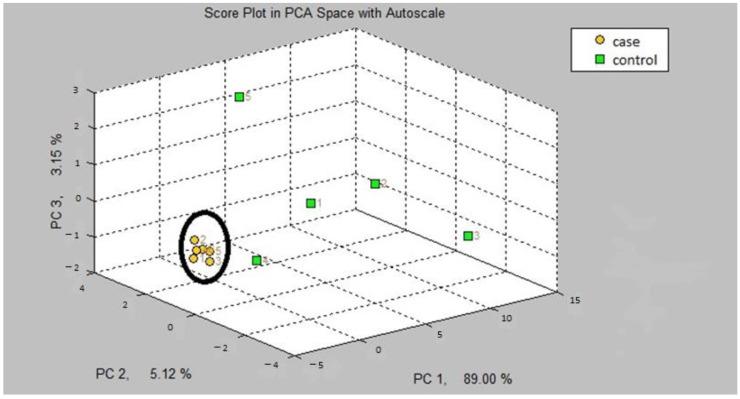
Three-dimensional PCA plot of model A.

**Figure 3:**
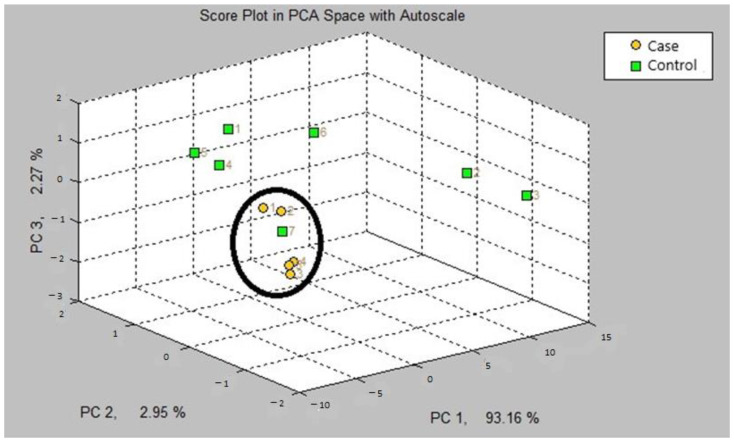
Three-dimensional PCA plot of model B.

The first step in our study was the internal validation step of the two training models. In the two models, asthmatic patients had distinct breath prints from controls. Figure 1 shows the breath sample fingerprint provided by e-nose for a patient with asthma. Table 3 contains the values of CVV, CVA and M-distance obtained through canonical discriminant analysis, while Figure 2 and Figure 3 show the three-dimensional PCA plots.

### 3.2. External Validation

After applying the training models, A and B, the subjects from the external validation set were classified. We evaluated training models based on their accuracy, sensitivity, specificity, positive predictive value and negative predictive value. The results are shown in Table 4. No statistical significance was found in the uncorrected chi square Pearson test, Yates test, Mantel Haenszel test and Fisher test.

### 3.3. Breath Prints’ Reproducibility

Five asthma patients and five controls were used to evaluate the breath prints’ reproducibility. Each subject gave a second breath print at a distance of five minutes from the first breath print. Pearson’s correlation coefficient was calculated for every pair of breath prints. In 8 of the 10 subjects, the coefficient was greater than or equal to 0.8, and the *p*-value was 0.01. The results are presented in Table 5.

## 4. Discussion

The diagnostic performance of the e-nose in pediatric asthma was evaluated. There was no difference between asthmatic and control groups when assessing for variations due to sex and living environment. It is important to note that patients with asthma were younger than the control patients in model A. Matched groups by age is not recommended by Dragonieri et al., taking into consideration that breath prints are not influenced by age [30], but their study was performed on adults and differences regarding asthma profiles can appear with age groups [13]. On the other hand, the living environment, especially smoking status, could influence the breath prints [31]. We did not evaluate smoking status or passive smoking in our study. Regarding the medication, the use of inhaled corticosteroids can influence the composition of exhaled breath [13]. For this reason, we used the breath air collection protocol proposed by Dragonieri et al., in which the patients are not allowed to take inhaled corticosteroids 12 h before the test. Their results showed no differences between the breath profiles of patients with severe asthma taking inhaled corticoids and long-acting bronchodilators as chronic treatment and patients with mild asthma, which were free from corticosteroids, suggesting that drug usage is not a major determinant of exhaled breath prints [17].

Regarding the statistical analysis, we applied the frequently used method of double validation (PCA and CDA).

In the first step of the study, the internal validation of both models A and B could adequately discriminate patients with asthma from the controls. We obtained a high cross-validation accuracy value of 90% for model B and 63.63% for model A for the offline program. The M-distance was equal to or more than 3 for both models.

Our results are comparable with the results found in the literature. Dragonieri et al. obtained a model to discriminate mild asthmatic patients from healthy young subjects with a CVA of 100% and an M-distance of 5.32. Severe asthmatics were distinguished from healthy controls with a CVA of 90% and an M-distance of 2.7 [17]. The same author obtained a CVA of 85.7% in a different study for a breath analysis model to discriminate between asthmatic patients and asthmatic patients with associated allergic rhinitis [9]. In contrast with these studies, we had a single group of asthmatic patients, the majority with partially controlled asthma, not taking into account severity of the disease. Atopy was present in all our patients from model B, where we obtained the most accurate discrimination.

In the field of pediatric asthma, studies regarding breath print analysis by e-nose are limited. The first one reported a sensitivity of 74% and a specificity of 91% for distinguishing between asthma patients and controls, with better results when discriminating between asthma and cystic fibrosis [10]. The second one reported weak cross-validation values for discriminating between asthma and controls, with a correct classification between 65 and 70% depending on the model [7]. The most recent studies, published in 2020, reported that e-nose could discriminate between controls and controlled asthma, between partially controlled and uncontrolled asthma groups with a sensitivity and specificity of 79% and 84% [14], and between patients with atopic asthma and non-atopic asthma [9]. In our study, accuracy, sensitivity and specificity were 64%, 77% and 50%, respectively, for model A and 58%, 66% and 50%, respectively, for model B.

The second objective of the study was an external validation of the two models. The diagnostic accuracy of the models in the external validation phase was modest and smaller than the accuracy obtained in the first phase. These differences were also highlighted by Leopold et al. [32]. As shown in Table 4, both models were more sensitive and less specific. The confidence intervals for the evaluated parameters were wide and, therefore, imprecise. However, our study is one of the few studies with an external validation component. Leopold et al. have pointed out that only 15% of the studies on breathomics have an external validation component [32]. In the field of asthma, there are few studies with both internal and external validation, and these were conducted on adults and on larger patients groups [9,24,33]. The study of Farraia et al. included a cohort of pediatric and adult patients with asthma-like symptoms, and with the use of an e-nose, they showed a very good identification of patients with severe asthma; the authors mention that the validation set contained few participants, which made it difficult to observe statistically significant differences between the groups [33]. Most recently, Abdel-Aziz et al. used a large study cohort, including adults and children with asthma, and demonstrated by internal and external validation that e-nose could identify the presence of atopy in asthmatic patients and could be used for asthma phenotyping [11].

Last but not least, we achieved the third objective of the study. Between paired fingerprints of the same subject, there were no significant differences. We obtained very good associations for 8 of the 10 pairs of breath prints. E-nose presents good reproducibility for a short period of time. Reproducibility was evaluated previously within-day and between-day [12,34].

As shown by studies using gas chromatography time of flight mass spectrometry, VOC analysis can differentiate preschool asthmatic children from transient wheezing children [8]. Future directions for this topic could be the external validation of the e-nose technique for identifying asthma in children under the age of five with recurrent wheezing, building asthma diagnosis models, which could permit earlier management. Recently, Rutjes et al. built a prediction model for asthma diagnosis of a group of school-aged, rhinovirus-positive, preschool wheezing children, obtaining a sensitivity of just under 80% but a relatively low specificity of 55% [15].

One limitation of our study was the small sample size, which can explain the limited discriminative power of the e-nose in this study. Our results require confirmation with a larger sample size. In our study, we included children older than five years for a better accuracy of asthma diagnosis based on GINA consensus; for children under five years old, breath air collection is possible [35], but to test the diagnostic accuracy of e-nose in this age group, the result should be confirmed by other diagnostic methods, such as chromatography or spectrometry. However, to our knowledge, this is one of the first studies that incorporates the external validation of the models built to identify pediatric asthmatic patients using e-nose breath prints analysis [11,32]. Another limitation that we identified was the control group. We noticed that the breath prints of asthmatic patients were much closer compared to the breath prints of controls. This is not surprising, taking into consideration that the controls were patients with other chronic non-respiratory diseases. It has been emphasized that breath prints are influenced by the comorbidities of the subjects [16]. The results could be also influenced by the age group difference in model A. Since the breath air composition can be influenced by multiple external and internal factors, in future research studies we should evaluate the breath prints of healthy children and identify differences regarding the age group, environmental factors, lifestyle and diet.

## 5. Conclusions

In conclusion, we would like to emphasize the need for a standard protocol for collecting breath samples in pediatric patients. In the absence of such a protocol, the results of different studies are difficult to analyze. Even though our study was performed on a small group of patients, the discriminative power of the models was satisfying. This could be improved by models based on more breath prints and better-defined groups. Additionally, it is important to keep in mind the need for external validation of the models. External validation provides valuable information on the discriminative power of the e-nose breath analysis, which could become a routine investigation in every day clinical practice, making the diagnostic process easier, reducing costs and helping the therapeutic decision.

## Figures and Tables

**Table 1:** Distribution of the patients and controls by age, sex and environment.

		Asthma	Control	*p*-Value
Training model A				
- Number (%)		6 (55)	5 (45)	-
- Age (years) ^a^		8.16 ± 3.48	14 ± 2.23	0.01
- Sex	M-number (%)	3 (50)	2 (40)	0.43
	F-number (%)	3 (50)	3 (60)	
- Environment	U-number (%)	6 (100)	2 (40)	0.06
	R-number (%)	0	3 (60)	
Training model B				
- Number (%)		5 (42)	7 (58)	-
- Age (years) ^a^		10 ± 2.54	12.85 ± 3.53	0.15
- Sex	M-number (%)	3 (60)	4 (57)	0.44
	F-number (%)	2 (40)	3 (43)	
- Environment	U-number (%)	5 (100)	3 (43)	0.07
	R-number (%)	0	4 (57)	
External validation set				
- Number (%)		9 (53)	8 (47)	-
- Age (years) ^a^		10.6 ± 5.01	12.25 ± 4.39	0.65
- Sex	M-number (%)	6 (66)	7 (87.5)	0.28
	F-number (%)	3 (34)	1 (12.5)	
- Environment	U-number (%)	7 (78)	3 (37.5)	0.16
	R-number (%)	2 (22)	5 (62.5)	

^a^ mean ± standard deviation, M—male, F—female, U—urban, R—rural.

**Table 2:** Clinical characteristics of asthmatic patients.

	Model A	Model B	External Validation Set
Family history of asthma (%)	50	40	70
History of respiratory infections (%)	66	20	80
Atopy (%)	66	100	90
History of bronchiolitis (%)	83	40	70
Symptoms onset (age in years) ^a^	3.8 ± 2.6	7.4 ± 2.7	6.7 ± 4.1
Medication			
- Intermittent low-dose ICS	1	1	1
- Low-dose ICS	2	3	4
- Medium-dose ICS	1	0	2
- Low-dose ICS + LTRA	2	1	2
Asthma severity (O/C/PC/UC)	1/0/5/0	1/1/3/0	0/3/3/3
Eo/Μl ^a^	580 ± 286	1048 ± 490	607 ± 452
Total IgE (IU/mL) ^a^	471 ± 519	404.6 ± 420	361 ± 448
Positive specific IgE for airborne allergens (%)	66	60	60
FEV1 ^a^ (% from estimated value)	83.8 ± 13	95.1 ± 10.3	90.2 ± 15
FEV1/FVC ^a^	82.4 ± 7.1	93.3 ± 8.4	90.1 ± 12

^a^ = mean ± standard deviation; Eo—eosinophils; O—at onset; C—controlled; PC—partially controlled; UC—uncontrolled.

**Table 3:** Diagnostic performance of the training models—internal validation.

	Model A	Model B
Online CVV ^1^ (%)	72.72	100
Offline CVA ^2^ (%)	63.63	90
Number of PC	4	4
M-distance	3.13	5.55

^1^ CVV—cross-validation value, ^2^ CVA—cross-validation accuracy.

**Table 4:** Diagnostic performance of the training models in the external validation phase.

	Model A	Model B
Accuracy (%) (IC)	64 (42–87.4)	58 (35.4–82.2)
Sensitivity (%) (IC)	77 (44.1–94.3)	66 (35.1–88)
Specificity (%) (IC)	50 (21.7–78.3)	50 (21.7–78.3)
PPV (%) (IC)	63 (35.2–92.1)	60 (29.6–90.4)
NPV (%) (IC)	66 (28.9–100)	57 (20.5–93.8)

PPV—positive predictive value; NPV—negative predictive value, CI—confidence interval.

**Table 5:** Breath prints’ reproducibility.

Subject	r	*p*-Value
Control 1	0.65	<0.01
Control 2	0.80	<0.01
Control 3	0.82	<0.01
Control 4	0.72	<0.01
Control 5	0.86	<0.01
Asthmatic1	0.84	<0.01
Asthmatic 2	0.83	<0.01
Asthmatic 3	0.91	<0.01
Asthmatic 4	0.80	<0.01
Asthmatic 5	0.89	<0.01

R—Pearson’s correlation coefficient.

## Data Availability

The data presented in this study are available on request from the corresponding author.
